# Rotationally Resolved Electronic Spectroscopy of UO_2_

**DOI:** 10.1021/acs.jpclett.6c00279

**Published:** 2026-02-21

**Authors:** Jiande Han, Jiayue Lin, Michael C. Heaven

**Affiliations:** Department of Chemistry, 1371 Emory University, Atlanta, Georgia 30322, United States

## Abstract

Rotationally resolved electronic spectra have been recorded for gas-phase UO_2_. Analysis of the rotational fine structure confirms that the ground state is X^3^Φ_2u_, derived from the O^2–^U^4+^(5f7s)­O^2–^ electronic configuration. All transitions observed below 14500 cm^–1^ were assigned to the [11.51]­3_g_ ↔ X^3^Φ_2u_ transition, which correlates with the metal-centered 5f7p ↔ 5f7s electron promotion. The symmetric stretch and bending vibrational modes were active in the spectrum. Ground-state vibrational fundamentals of 853(5) and 133(5) cm^–1^ were determined along with a vibrationally averaged bond length of 1.781 Å. Spectra for the [17.68]­4_g_ ↔ X^3^Φ_3u_ transition exhibited the same lower state vibrational frequencies, confirming that the X^3^Φ_3u_ state also arises from the 5f7s configuration. Fluorescence decay lifetimes for the excited states were in the range of 1.5–4.7 μs, values that were anomalously long for electric dipole-allowed transitions. The long lifetimes are attributed to extensive mixing with a dense background of vibronic dark states.

## Graphical abstract

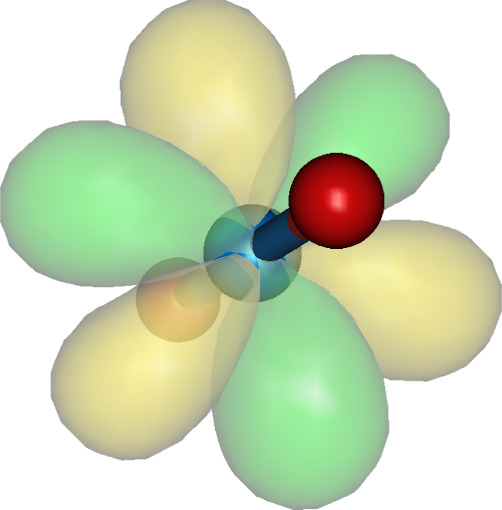

Uranium dioxide is a technologically important material that is widely used to fuel nuclear reactors for electrical power generation. It is a prototypical actinide-containing molecule that has been the focus of many theoretical and experimental investigations. Spectroscopic studies of UO_2_ initially used rare gas matrix isolation with infrared absorption measurements of the vibrational frequencies.^1–6^ ^16^O/^18^O isotopic substitution was used to show that the molecule has a linear, centrosymmetric equilibrium structure (D_∞h_). Typically, the cryogenic matrix hosts Ne and Ar are considered to be minimally perturbing such that the properties of the guest molecules are close to those of the gas phase. However, Andrews and co-workers^5,6^ found that the frequency of the UO_2_ antisymmetric stretch was dramatically increased by going from Ar to Ne matrices, with Δ*G*_1/2_ values of 776.0 and 914.8 cm^–1^, respectively. Spin-free density functional theory (DFT) calculations provide a reasonable interpretation for this anomaly. Zhou et al.^6^ found that the ground state was formally O^2–^U^4+^(5f7s)­O^2–^, ^3^Φ_2u_ with an antisymmetric stretch vibrational frequency of 931 cm^–1^. DFT also predicted the presence of a O^2–^U^4+^(5f^2^)­O^2–^, ^3^H_4g_ state at an energy of 1920 cm^–1^, with an antisymmetric stretch frequency of 814 cm^–1^. Andrews et al.^7,8^ speculated that due to differential matrix shifts, the ground state of UO_2_ switched from ^3^Φ_2u_ in Ne to ^3^H_4g_ in Ar. Subsequent CCSDT calculations were used to show that the interactions between UO_2_ and a surrounding equatorial ring of five Ar atoms (D_5h_ structure) was strong enough to reorder the low-energy electronic states of UO_2_ relative to the gas phase.^8^ One of the implications of these and related studies was that, for U-containing molecules, rare gas matrices could not be treated as minimally perturbing hosts. A further complication was discovered when the electronic spectrum of UO_2_ was examined in an Ar matrix.^9^ The similarity of the matrix and gas-phase spectra indicated that the ^3^Φ_2u_/^3^H_4g_ state ordering had not been reversed by interactions with Ar.

Electronic spectra for gas-phase UO_2_ were reported by Han et al.^10^ This study used resonantly enhanced two-photon ionization (RE2PI) to observe vibronic bands in the 17400–31850 cm^–1^ range. Among other properties, these measurements provided the ground-state bending frequency (120(10) cm^–1^) and the term energy of the first electronically excited state (360(10) cm^–1^). The latter was also reported at 347(5) cm^–1^, based on a high-resolution photodetachment spectrum of UO_2_^–^.^11^

A surprising limitation of the RE2PI experiment was that the rotational structures of the vibronic bands could not be resolved, despite the use of jet expansion cooling and a tunable laser with a sufficiently narrow line width. Han et al.^10^ speculated that a high density of vibronic states was responsible for the spectral congestion. In this model each spectrally bright excited state is embedded in a background of dark states that are rendered observable by perturbative intensity borrowing.^12,13^ Evidence of state mixing was provided by a two-color pulsed-field ionization zero kinetic energy photoelectron (PFI-ZEKE) study.^14^ This technique revealed the ionization threshold and the low-energy states of the UO_2_^+^ cation. The first laser was used to excite a transition of neutral UO_2_. Scanning the wavelength of the second laser then yielded a spectrum for UO_2_^+^ in which the intensity distribution was expected to follow the Franck–Condon factors (FCF) for the vibrational overlap between the excited state of the neutral molecule and the final states of the ion. As ground-state UO_2_, optically excited UO_2_, and UO_2_^+^ all have linear symmetric structures, a near-diagonal FCF distribution was anticipated. Instead, a highly congested pattern of energy levels was observed with very long progressions of the bending modes built on the symmetric stretch mode and a low-energy electronically excited state. This violation of the Franck–Condon principle was taken as evidence of the high density of states accessed by exciting the neutral molecule transition.

Resolution of the rotational substructure of UO_2_ spectra is essential for the reliable determination of the geometry and electronic state symmetries.^15,16^ An obvious strategy for obtaining these data is to search for lower-energy transitions where the density of the background states is reduced. This was the primary motivation for the present work. Han et al.^10^ used mass-resolved ion detection for their RE2PI measurements. That technique was chosen to reject signals from other species in the gas-phase sample including U, UO, and uranium oxide clusters. For this study, we used two-dimensional fluorescence excitation spectroscopy with time-gated fluorescence detection (2d-LIF) to disentangle the overlapping band systems.

To facilitate the presentation and discussion of the UO_2_ spectrum, it will be helpful to define the chosen system of notation. The vibrational modes that were optically active were the symmetric stretch and the bending modes (labeled as *v*_1_ and *v*_2_). Excited states of the bending mode are also distinguished by their vibrational angular momentum, given by the unsigned quantum number *l*. For the observed bands, the lowest *l*-values were active (e.g., *l* = 1 for *v*_2_ = 1 and *l* = 0 for *v*_2_ = 2). Vibronic transitions are labeled by *N*_*v*_″__^*v* ^′^^ where *N* is the vibrational mode number and *v*′ and *v*″ are the excited and ground state vibrational quantum numbers. For example, 2_1_^1^ indicates a transition in which one quantum of the bending mode is excited in both the ground and electronically excited states. Electronically excited states above 10000 cm^–1^ are labeled as [T_0_/10^3^]­Ω, where T_0_ is the term energy in wavenumbers and Ω is the unsigned projection of the electronic angular momentum along the internuclear axis.

Our search for low-energy transitions covered the regions 11370–11675, 11970–12245, and 12675–13715 cm^–1^. The gaps in coverage were due to limitations of our Raman-shifted dye laser system. Bands at energies above 14000 cm^–1^ were examined using the dye laser without Raman shifting, yielding data with somewhat better signal-to-noise ratios.

Several groups of bands were observed that exhibited easily recognized sequences of the bending mode. Figure 1 shows a typical example. The bands near 12900 cm^–1^ are the 1_0_^2^, 1_0_^2^ 2_1_^1^, and 1_0_^2^2_2_^2^ sequence. The 1_0_^2^2_0_^2^ band is evident near 13125 cm^–1^ (justification for the symmetric stretch assignments is given below). This same vibronic structure pattern was observed in our previous study^10^ of the UO_2_ bands near 17500 cm^–1^.

**Figure 1:**
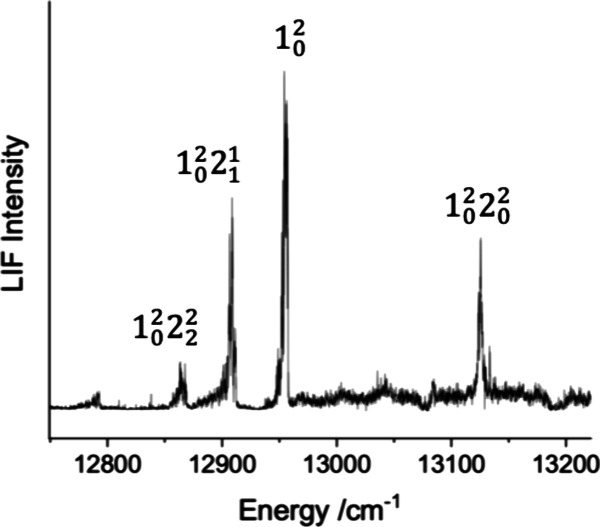
Bending mode vibronic structure associated with the 1_0_^2^ transition of [11.51]­3_g_-X^3^Φ_2u_.

Table 1 lists the vibronic transition energies and assignments determined in the present study. Rotational structure was resolved for the 1_0_^*v* ^′^^ bands with the best quality data obtained for the 1_0_^4^ transition at 14394 cm^–1^. The rotational structure of this band, along with a PGOPHER^17^ fitted simulation, is shown in Figure 2. To obtain this trace, the fluorescence detection wavelength was scanned synchronously with the excitation wavelength. The simple P/Q/R-branch rotational structure of Figure 2 shows that the equilibrium structure was of D_∞h_ symmetry for both the ground and excited states. Furthermore, the first lines of the P and R branches can be clearly identified as P(4) and R(2). As the total angular momentum J cannot be smaller than Ω, this demonstrates that the upper and lower state Ω values were 3 and 2, respectively. Since the study by Zhou et al.,^6^ electronic structure calculations have consistently predicted an Ω″ = 2_u_ ground state,^8,18–23^ so the excited state is assigned to Ω′ = 3_g_. The simulation shown in Figure 2 used an approximate rotational temperature of 10 K and defined rotational constants of B_0_″ = 0.1660(5) and B′ = 0.1589(5) cm^–1^. This value for B_0_″ was fixed in the fitting for bands recorded using the Raman-shifted dye laser.

**Table 1:** Molecular Constants Derived from the [11.51]­3_g_-X^3^Φ_2u_ Transition of UO_2_

Assignment	*v*_0_ ^a^	B′	ΔE_sequence_ ^b^	ΔE_ss_^′^	τ^d^/μs
2_2_^2^	11406		–104		
2_1_^1^	11458		–51		
0_0_^0^	11509.0(2)	0.1593(8)	0	0	2.5(2)
2_0_^2^	11662.0		153		
					
1_0_^1^2_2_^2^	12127		–103		
1_0_^1^2_1_^1^	12178		–53		3.2(2)
1_0_^1^	12230.6(2)	0.1576(8)	0	722	4.7(2)
					
1_0_^2^2_2_^2^	12863		–92		
1_0_^2^2_1_^1^	12909		–46		
1_0_^2^	12955.0(2)	0.1567(8)	0	724	1.5(2)
1_0_^2^2_0_^2^	13125		170		
				720^c^	
1_0_^4^2_1_^1^	14359		–35		
1_0_^4^	14394.1(1)	0.1589(5)	0		3.3(2)

a Energies in cm^–1^ units. Errors are ±1 cm^–1^ unless otherwise indicated.

b Displacement relative to the 0_0_^0^ or 1_0_^*v* ^′^^ level.

c This value is half the interval between 1_0_^2^ and 1_0_^4^.

d Fluorescence decay lifetime.

**Figure 2:**
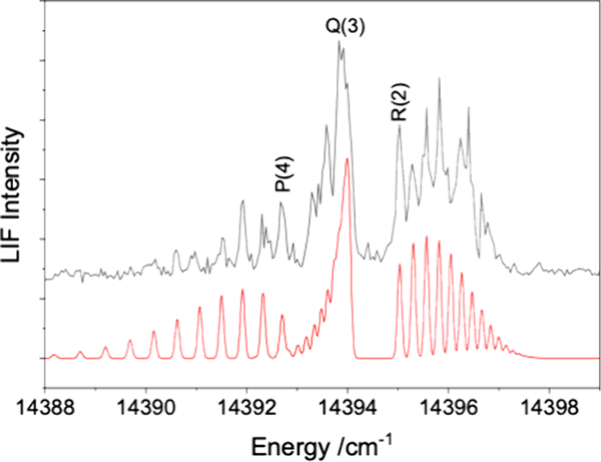
Rotational structure of the [11.51]­3_g_-X^3^Φ_2u_ 1_0_^4^ band. The lower trace is an optimized simulation generated by using the PGOPHER software package.

Starting from 11509 cm^–1^ a progression of 1_0_^*v* ^′^^ bands was identified with *v*′ = 0–4. Of this series the 1_0_^3^ band was weak but present with an intensity contour maximum at 13670 cm^–1^. Fitting to the band origins yielded vibrational constants of ω_e_^′^ = 724(3) and ω_e_x_e_^′^ = 0.7(5), with the latter being of marginal significance. The rotational structures for the *v*′ = 0, 1, and 2 bands were consistent with the [11.51]­3_g_-X^3^Φ_2u_ electronic transition, as noted above.

The bending sequence bands yielded a consistent ground state vibrational interval of ΔG″_1/2,bend_ = 130(5) cm^–1^. The upper state bending vibrational sequence intervals 1_0_^*v* ^′^^ – 1_0_^*v* ^’′^^ 2_1_^1^ were dependent on the symmetric stretch state, with values of 51, 53, 46, and 35 cm^–1^ for *v′* = 0, 1, 2, and 4. This trend indicated that the excited state vibrational interval slightly increased with increasing *v*′ (ΔG′_1/2,bend_ = 79, 77, 84 and 95, errors of ±3 cm^–1^).

The ground state symmetric stretch fundamental was determined from a 2d-LIF spectrum produced by excitation of the 1_0_^4^ band at 14394 cm^–1^. Emission bands were observed at the resonant frequency and at 13541 cm^–1^. The intensity pattern suggested that there was a progression that ran to energies below 13000 cm^–1^, but this could not be verified as the detector response fell rapidly below this energy. The vibrational interval determined by the two emission features was ΔG″_1/2,ss_ = 853(5) cm^–1^. Emission bands associated with excitation of the ground state bending mode or radiative relaxation down to the X^3^Φ_3u_ state were below the noise level.

Han et al.^10^ reported vibronic bands that were ascribed to a [17.86]­4_g_-X^3^Φ_3u_ transition, where the X^3^Φ_3u_ state was just 360 cm^–1^ above X^3^Φ_2u_. We revisited the 17499 cm^–1^ 0_0_^0^ origin band of the [17.86]­4_g_-X^3^Φ_3u_ transition using 2d-LIF and obtained unexpected results. The fluorescence decay curve generated by exciting at the band contour maximum exhibited an approximately exponential decay with a lifetime of 2.2(1) μs. Consequently, 2d-LIF spectra for the 0_0_^0^ band were recorded by using a 1 μs fluorescence integration window. In contrast to the earlier RE2PI spectrum, the fluorescence detected spectrum displayed partially resolved rotational structure. The PGOPHER^17^ software package was used to simulate the rotational structure with the lower state rotational constant fixed at B″ = 0.1660 cm^–1^. This choice was based on the expectation that the properties of the X^3^Φ_3u_ and X^3^Φ_2u_ states would be closely similar. The rotational structure was found to be consistent with the [17.86]­4_g_-X^3^Φ_3u_ assignment and yielded an upper state rotational constant of B′ = 0.157(1) cm^–1^.

Figure 3 shows a dispersed LIF spectrum (cut through the 2d-LIF data along the emission axis) resulting from excitation of the [17.86]­4_g_-X^3^Φ_3u_ transition at 17500 cm^–1^. Three bands were observed with an intensity pattern that was consistent with emission from the zero-point level of the excited state. Radiative emission down to the X^3^Φ_2u_ state was not seen as this is forbidden by the ΔΩ = 0, ±1 selection rule. The average interval between the three observed bands, 849(5) cm^–1^, was statistically indistinguishable from the X^3^Φ_2u_ vibrational interval of 853(5) cm^–1^.

**Figure 3:**
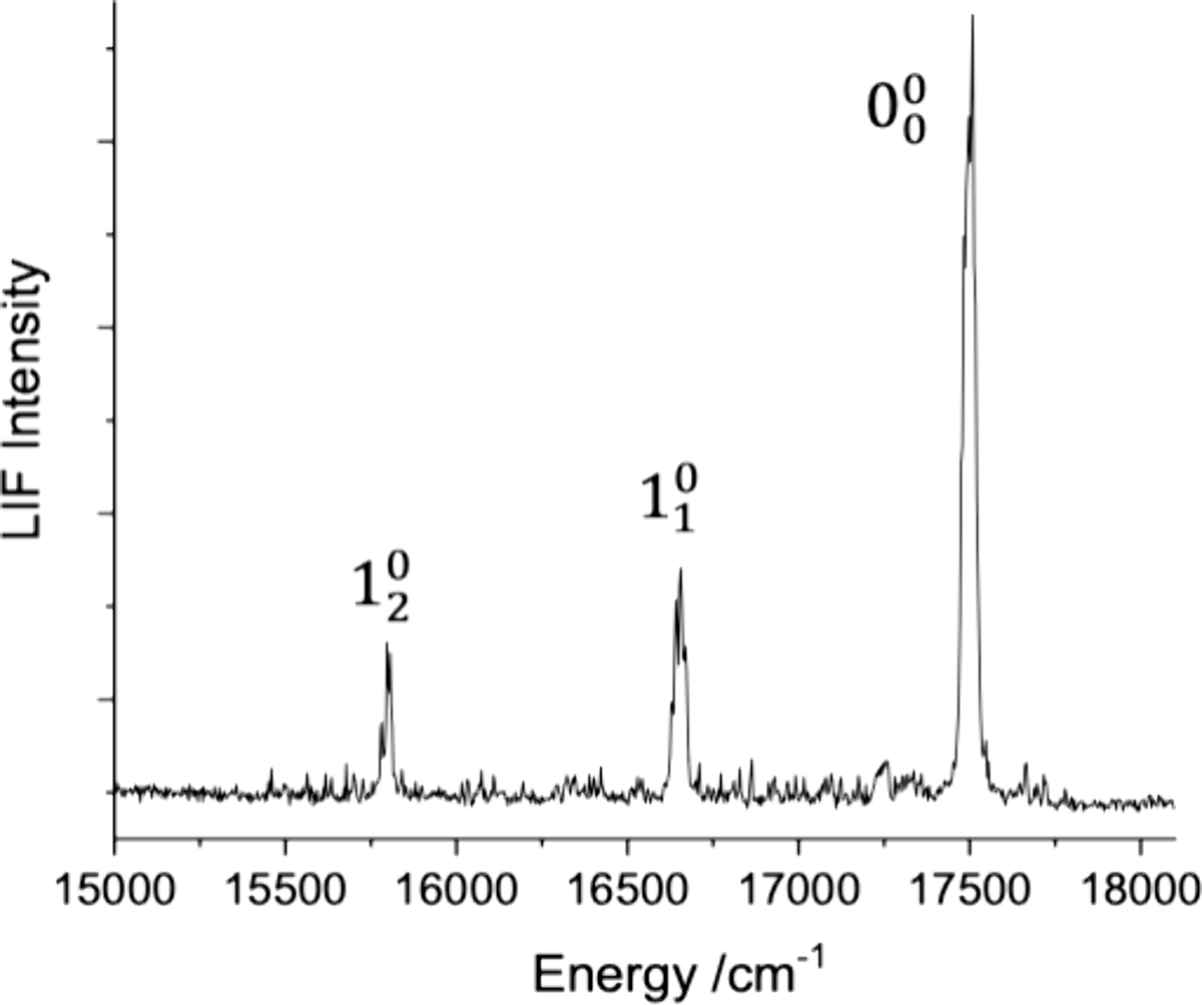
A dispersed fluorescence spectrum resulting from excitation of the [17.68]­4_g_-X^3^Φ_3u_ 0_0_^0^ band at 17500 cm^–1^.

An interesting characteristic of the [17.86]­4_g_ 0_0_^0^ state was the dependence of the fluorescence decay lifetime on rotational excitation. Excitation of different features of the R-branch produced decay curves with lifetimes ranging from 1.5 to 4.7 μs, and several showed significant deviations from single exponential decay. There were no obvious correlations between these variations. Fluorescence decay curves for selected vibronic bands of the [11.51]­3_g_-X^3^Φ_2u_ transition were also examined. These showed deviations from signal exponential decay, but we approximated the kinetics by fitting to single exponentials. The results are collected in Table 1.

The ground state rotational constant derived from the present measurements defines a vibrationally averaged bond length of R_0_ = 1.781 Å. Electronic structure calculations have predicted values for the ground state equilibrium bond length of R_e_ = 1.766–1.816 Å.^16–21^ The R_e_ value will be slightly shorter than R_0_, but it is clear that the bond length was reasonably well predicted by using a range of computational models. Comparisons of measured and calculated vibrational frequencies for low energy states of UO_2_ are complicated by the fact that most of the experimental data was obtained using matrix isolation techniques. The studies carried out by Gabelnick et al.^1,2^ used Ar matrices that Andrews and co-workers subsequently demonstrated to be a strongly perturbing host.^6–8^ Data recorded from a Ne matrix provided only the antisymmetric stretch frequence (ΔG″_1/2,as_ = 914.8 cm^–1^).^6^ The DFT calculations of Zhou et al.^6^ and Li et al.^8^ gave predictions that were in good agreement with the antisymmetric stretch frequency (931 and 919 cm^–1^, respectively). This indicates that the frequencies predicted for the symmetric stretch (874 and 856 cm^–1^) should be reasonably reliable. The present gas phase measurement of the symmetric stretch frequency agrees with the value reported by Li et al.^8^ within the experimental error range.

The DFT calculations did not include spin–orbit coupling; therefore, the agreement with experiment suggests that the characteristics of the potential energy surfaces near the equilibrium geometry are not significantly influenced by this interaction. As the X^3^Φ_2u_ and X^3^Φ_3u_ states both arise from the metal-centered 5f7s electronic configuration it is not surprising that they exhibit similar vibrational constants. In our previous study^10^ of UO_2_ we reported bending frequencies of 121(10) and 135(10) cm^–1^ for the X^3^Φ_2u_ and X^3^Φ_3u_ states. A reanalysis that combined the present and previous data showed that both states had a common bending frequency of 133(5) cm^–1^.

All vibronic transitions observed at energies below 14500 cm^–1^ could be assigned to a single [11.51]­3_g_-X^3^Φ_2u_ band system. High-level theoretical calculations that included spin–orbit coupling predicted that the most intense transitions would be associated with the metal-centered 5f7p ← 5f7s electron promotion. Tyagi^19^ and Gagliardi et al.^18^ found that the lowest energy state of the 5f7p configuration was 3_g_. The calculations of Gagliardi et al.^18^ indicated that the 3_g_ and 4_g_ states reported here had leading eigenvector components of 5fϕ^1^7pπ^1^, ^3^Γ_3g_ and 5fϕ^1^7pπ^1^, ^3^Γ_4g_, respectively. Oscillator strengths for the 3_g_-X^3^Φ_2u_ transition were *f* = 0.1047,^19^ 0.0595.^18^ The term energy for the 3_g_ state was predicted to be 13182 ^19^ or 13411 cm^–1^.^18^ Infante et al.^20^ used the Dirac–Coulomb intermediate Hamiltonian Fock-space coupled cluster method for their calculations. They found that the 3_g_ state derived from 5f7p was at a substantially higher energy (21147 cm^–1^) with a 3_g_-X^3^Φ_2u_ oscillator strength of 0.1686 for the largest basis set examined. Their lowest 3_g_ state at 15502 cm^–1^ was derived from the 7s6d configuration and had no oscillator strength for the transition from X^3^Φ_2u_. A consistent feature of the three computational studies was that the term energy for the optically active 3_g_ state was always above the lowest energy vibronic band observed. This was used as the basis for the tentative 0_0_^0^ assignment of the 11509 cm^–1^ band.

The symmetric stretch progression of the [11.51]­3_g_ state was easily identified in the LIF spectrum. The average vibrational interval of 724 cm^–1^ was smaller than that of the ground state, signaling a change in the electronic configuration on excitation. This decrease in the vibrational frequency was accompanied by an increase in the bond length of 0.039 Å. Hence, the observation of a progression in the symmetric stretch vibrational mode was consistent with the changes in the frequency and bond length. The influence of electronic excitation on the bending frequency is difficult to anticipate, but the result was that the frequency was substantially reduced on excitation to the [11.51]­3_g_ state.

The calculations of Tyagi^19^ and Gagliardi et al.^18^ assigned the upper state of the previously reported [17.86]­4_g_-X^3^Φ_3u_ transition to the 5f7p configuration. Hence, it was expected that the molecular constants for the [11.51]­3_g_ and [17.86]­4_g_ states would be similar. The fundamental bending frequencies for the upper states were in agreement, to within the measurement errors (71 ± 10 cm^–1^). The vibronic band analyses proposed in ref 10 did not consider excitation of the stretching modes, leaving some features without assignments. Given the insights gained from the present study, it now appears that the band at 18227 cm^–1^ is [17.86]­4_g_-X^3^Φ_3u_ 1_0_^1^, yielding a symmetric stretch fundamental frequency of ΔG′_1/2,ss_ = 728 cm^–1^.

The fluorescence decay lifetimes of the [11.51]­3_g_ and [17.86]­4_g_ states appeared to be anomalously long. Theoretical calculations^18–20^ predict that the absorption spectrum will be dominated by the allowed transitions derived from the 5f7p ← 5f7s electron promotion. The oscillator strengths for these transitions were found to be in the *f* = 0.05–0.11 range. These values, combined with the electronic transition frequencies, yielded spontaneous decay lifetimes of 45–190 ns. The >1 μs lifetimes observed suggest that extensive mixing with dark background states is responsible for the inconsistency. Mechanisms for the lengthening of lifetimes have been discussed by Douglas,^12^ and UO_2_ fits the details for the type C and D models (NO_2_ provides an excellent example of these effects). For UO_2_ we previously attributed the poor resolution of the structures within the vibronic bands to the mixing with background states.

An unexpected discovery of the present work was that spectra recorded using LIF with wavelength-selected, time-integrated fluorescence detection were significantly better resolved than the previous two-color RE2PI results. The laser intensities used for both techniques were comparable; therefore, it is unlikely that the resolution was limited by power broadening of the resonant transition. The RE2PI measurements were conducted using two 10 ns laser pulses that were overlapped in space and time. It is possible that the simultaneous presence of two radiation fields increased state mixing. Further studies of the time-evolution of the excited states are needed to understand the factors that determined the intrinsic line widths.

In summary, low energy vibronic transitions of gas phase UO_2_ have been characterized by using one- and two-dimensional LIF techniques. Bands that were observed in the 11400–14400 cm^–1^ range could all be assigned to a [11.51]­3_g_-X^3^Φ_2u_ transition. Theoretical calculations show that this transition is derived from the electric dipole-allowed 5f7p ← 5f7s electron promotion. The symmetric stretch and bending vibrational modes were active in both the absorption and the emission spectra. Rotational resolution was achieved for a subset of bands. Analyses of these data confirmed that the equilibrium structure was of D_∞h_ symmetry, and that the electronic ground state has an electronic angular momentum projection of Ω = 2_u_. Vibrational frequencies for the X^3^Φ_2u_ and X^3^Φ_3u_ states were found to be the same within the measurement errors. This agreed with the expectation that states arising from the 5f7s configuration would have very similar ro-vibrational constants. Overall, the theoretical predictions of Tyagi^19^ and Gagliardi et al.^18^ were found to be in reasonable agreement with the experimental results. One notable discrepancy was that the radiative lifetimes measured for the excited states were far larger than the values predicted by using oscillator strengths resulting from electronic structure calculations. We speculate that a high degree of state mixing is responsible for the anomalously long lifetimes.

## Experimental Methods

The apparatus used for these experiments has been described previously.^15,24^ Gas-phase UO_2_ was generated using a Smalley-type laser ablation nozzle.^25^ The second harmonic of a Nd/YAG laser was used to ablate the surface of a depleted uranium rod. The metal vapor was entrained in a carrier gas flow consisting of 2% O_2_ in Ar. Downstream of the ablation zone, the gas mixture was supersonically expanded through a 0.8 mm diameter orifice into a vacuum chamber. This cooled the UO_2_ rotational temperature down to the 10–20 K range.

The beam from a pulsed tunable dye laser (Lumonics HyperDye 300) was used to excite the gas jet approximately 10 cm downstream from the nozzle orifice. The laser beam traversed the gas expansion with an incidence angle of 90°. LIF was detected along an axis that was perpendicular to the plane defined by the gas jet direction and the laser beam. For 2d-LIF measurements, the fluorescence was collimated and focused through the entrance slit of a 0.67 m monochromator (McPherson model 207 with a 300 lines/mm diffraction grating). To record 2d spectra, the exit slit of the monochromator was replaced by a time-gated and intensified charge coupled device array (ICCD, Andor iStar DH320T-18U-63). In the visible range, an approximately 75 nm window of the dispersed fluorescence spectrum was viewed at a given laser excitation wavelength. Raman-shifting of the dye laser was used to access transitions at wavelengths greater than 740 nm. This was accomplished using a 1 m long cell filled with pure H_2_ at a pressure of 350 psia. The photon energy of the stimulated Raman output was downshifted by 4155.2 cm^–1^ relative to the input photon energy. The ICCD camera had a long wavelength cutoff near 740 nm. Consequently, longer-wavelength 1d-LIF spectra were recorded by using a cooled RCA C31034 photomultiplier tube. Long-pass optical filters were used to reduce the scattered laser light. Wavelength calibration of the laser was achieved by the simultaneous recording of the I_2_ B-X bands.

## Supplementary Material



## Notes

Transparent Peer Review report available (PDF)
