# Supplementary material for: Rotationally Resolved Electronic Spectroscopy of UO2
Source: J Phys Chem Lett. 2026 Feb 21;17(9):2661–5. doi: 10.1021/acs.jpclett.6c00279 (PMC12969359; doi:10.1021/acs.jpclett.6c00279)
Supplement: Supplementary file 1 [file jz6c00279_si_001.pdf]

jz-2026-00279r.R1

Name: Peer Review Information for "Rotationally resolved electronic spectroscopy of UO₂"

First Round of Reviewer Comments

Reviewer: 1

Comments to the Author

This study of the rotationally-resolved electronic spectra of gas-phase uranium dioxide is another valuable piece of work by Michael Heaven, a leader in high resolution electronic spectroscopy, especially that of actinide molecules. To reduce the clutter associated with background states, Heaven and his colleagues focused on low energy transitions, and used two-dimensional, laser induced fluorescence with temporal gating to distinguish between overlapping bands. I recommend that this paper be published as is.

Reviewer: 2

Comments to the Author

Review of "Rotationally resolved electronic spectroscopy of UO_2," by Jiande Han, Jiayue Lin, and Michael C. Heaven

In this manuscript, the authors report rotationally resolved vibronic spectra of two band systems of the triatomic UO_2 molecule along with dispersed fluorescence spectra. The spectra originate from the $X^3\Phi_{2u}$ ground state and its spin-orbit excited component, $X^3\Phi_{3u}$. Upper and lower state rotational constants are reported, which are used to obtain vibrationally averaged bond lengths for the $D_{\infty h}$ UO_2 molecule. Symmetric stretching and bending modes are active, both in the excitation and dispersed fluorescence spectra, allowing these vibrational frequencies to be determined for both upper and lower states. The rotational and vibrational constants are quite similar for the two lower states,

demonstrating that the $X^3\Phi_{2u}$ and $X^3\Phi_{3u}$ levels are spin-orbit components of the same Λ-S state. The ground state is assigned to an electronic configuration that places two unpaired electrons in the 5fφ and 7sσ orbitals, leading to the ground $\varphi^1\sigma^1$, $^3\Phi$ term, in agreement with theory. The observed band systems are the $[11.51]3_g \leftarrow X^3\Phi_{2u}$ and $[17.68]4_g \leftarrow X^3\Phi_{3u}$ systems, which correspond to a 7p ← 7s excitation. These band systems are in reasonably good agreement with previous computational work.

Although this molecule has been experimentally studied before through matrix-isolation experiments, those experiments had discrepancies on the ground state assignment, which have been clarified in this rotationally resolved gas phase study. The measured radiative lifetimes do not match computational results, but the proposed explanation for this discrepancy – that the excited levels are highly mixed with dark states lying in the same energy region – is highly plausible and is even expected. It is good to see the classic papers of Douglas (ref 12) and Freed and Nitzan (ref 13) cited in this context.

This manuscript is very well written and clear. The UO_2 molecule has been of great interest to computational chemists and uranium chemists for many years, and this work is by far the most definitive experimental investigation to date. The high level of interest in this species makes this study completely appropriate for publication in the Journal of Physical Chemistry Letters. It may not be obvious to the non-expert, but the high density of vibronic states in this "simple" molecule made this investigation far more difficult than is apparent. I strongly recommend acceptance of the paper after some minor issues, noted below, are addressed.

Recommended changes to the text:

1. In several places the authors refer to the "asymmetric stretching vibration" of UO_2 (page 2, lines 17, 23, 26; page 8, line 39). Since the normal mode in question changes sign upon reflection in the σ_h plane, this should be described as the "antisymmetric stretching vibration." I realize that 90% of chemists would describe this as the "asymmetric stretch", so I may be fighting a losing battle here, but I think the distinction between antisymmetric (changing sign when the symmetry operator acts on it) and asymmetric (literally, without symmetry) is important to maintain. The authors do use the term "antisymmetric" to describe this vibrational mode on page 8, line 35, but I think this should not be hyphenated, as "antisymmetric".
2. The authors identify the $[11.51]3_g$ and $[17.86]4_g$ states as arising from the $5f^17p^1$ configuration (referencing the work of both Tyagi and Gagliardi). I think it would be worthwhile to point out that Gagliardi's work shows that these states are dominated by

$5f\varphi^1 7p\pi^1$, $^3\Gamma_{3g}$ and $5f\varphi^1 7p\pi^1$, $^3\Gamma_{4g}$ character, respectively.

3. Page 9, lines 28-47, is a bit confusing to me. The calculation of Li *et al*. that is referenced in lines 32-35 needs clarification, as it isn't obvious that this is relevant to the excited states probed here. Li's calculation compares the $5f\varphi^1 7s\sigma^1$, $X^3\Phi$ ground state (calculated symmetric stretch frequency 856 cm^{-1}) to the $5f\varphi^1 5f\delta^1$, ^3H excited state (calculated symmetric stretch frequency 779 cm^{-1}) and the manuscript attempts to relate this to the excited state vibrational frequencies found for the $[11.51]3_g$ and $[17.86]4_g$ states in the present study. However, the excited states probed here arise from $5f^1 7p^1$ occupations and are much higher energy states than the $5f\varphi^1 5f\delta^1$, ^3H state, for which Gagliardi finds the 4_g level to lie at 3330 cm^{-1}. I would not expect states arising from a $5f^2$ configuration to have similar vibrational frequencies to those arising from a $5f^1 7p^1$ configuration. The compact nature of the 5f orbital is expected to shield the oxide ligands from the positive charge of the uranium nucleus more effectively than the diffuse 7s orbital, explaining why the $5f\varphi^1 7s\sigma^1$, $X^3\Phi$ ground state has a higher vibrational frequency than the calculated $5f\varphi^1 5f\delta^1$, ^3H excited state. However, I would expect the even more diffuse 7p orbital (particularly in its $7p\pi$ orientation) to be even less effective than the 7s orbital in shielding the oxide ligands from the uranium nuclear charge. Therefore, I would expect these excited states to have comparable or even higher vibrational frequencies than the ground state, perhaps approaching the vibrational frequencies of the cation, UO_2^+. In this connection, it would be useful to compare the excited state vibrational frequencies to those of UO_2^+, if those are known. In any case, I don't think this argument explains why the excited states probed here have a lower symmetric stretching frequency than the ground state.
4. Just as a side note, I'm a bit surprised that the $\Delta v=0, \pm 2, \pm 4$ rule is followed for the bending mode in these spectra. This indicates that there isn't significant vibronic interaction with nearby states that differ in Λ by ± 1. I remember that such interactions were very clearly seen in studies of NiCN by Anthony Merer.

Overall recommendation:

This spectroscopic investigation of UO_2 is of the highest caliber, regarding a molecule that has been of great interest to computational chemists and actinide chemists for many years. It should be accepted for publication in the Journal of Physical Chemistry Letters after the authors have responded to the comments offered above as they see fit. No further review is required.

Author's Response to Peer Review Comments:

February 11, 2026

Dear JPCL editor:

I have submitted a revised manuscript for the paper (jz-2026-00279r), "Rotationally resolved electronic spectroscopy of UO_2" for publication in the Journal of Physical Chemistry Letters. Our response to the referees' comments are as follows:Thanks to referee #1 who recommended publication as is. Referee #2 suggested helpful edits that have been adopted in the revised paper.

Comment 1. The referee recommended the change from "asymmetric" to "antisymmetric" throughout the paper. This has been implemented.

To address comment number 2 we have added the text "the 3g and 4g states reported here had leading eigenvector components of $5f\square^{1}7p\square^{1}$, $^{3}\square_{3g}$ and $5f\square^{1}7p\square^{1}$, $^{3}\square_{4g}$, respectively" on page 9.

Comment 3. The referee correctly notes that the comparison of the excited state vibrational frequencies associated with the $5f^2$ and 5f7p configurations is not justified. On page 9 we have deleted the text, "Li et al.[8] calculated the vibrational frequencies for the $X^{3}\square$ and ^{3}H states and obtained symmetric stretch frequencies of 856 and 779 cm^{-1}, respectively. In part, the implied change in the bond stiffness reflects the greater electrostatic repulsion experienced by the less polarizable $5f^2$ configuration. In addition to the electrostatic consideration, it appears that the 7p orbital may also contribute less to the bonding."

Comment 4 – We agree that obeyance of the $\square v_{bend}$=even selection rule is surprising, but the molecule has the last word.

1515 Dickey Drive, Emory University, Atlanta, Georgia, 30322
Departmental Phone: (404) 727-6585 Fax: (404) 727-6586 http://www.emory.edu/CHEMISTRY

Sincerely,

M. Heaven

Michael C. Heaven

Professor of Chemistry
